# BRAF and KRAS mutations in papillary thyroid carcinoma in the United Arab Emirates

**DOI:** 10.1371/journal.pone.0231341

**Published:** 2020-04-21

**Authors:** Suhail Al-Salam, Charu Sharma, Bachar Afandi, Khaled Al Dahmani, Ali S. Al-Zahrani, Amal Al Shamsi, Juma Al Kaabi

**Affiliations:** 1 Department of Pathology, College of Medicine & Health Sciences, United Arab Emirates University, Al Ain, United Arab Emirates; 2 Department of Internal Medicine, College of Medicine & Health Sciences, United Arab Emirates University, Al Ain, United Arab Emirates; 3 Endocrine Division, Tawam Hospital, Al Ain, United Arab Emirates; 4 Department of Medicine, Molecular Endocrinology Division, Alfaisal University, Riyadh, Saudi Arabia; 5 Department of Molecular Oncology, King Faisal Specialist Hospital & Research Center, Riyadh, Saudi Arabia; Universidade do Porto Faculdade de Medicina, PORTUGAL

## Abstract

**Background:**

Papillary thyroid carcinoma (PTC) is the most common malignant thyroid neoplasm comprising 80–90% of all thyroid malignancies. Molecular changes in thyroid follicular cells are likely associated with the development of PTC. Mutations in serine/threonine-protein kinase (*BRAF*) and Rat sarcoma viral oncogene homolog (*RAS*) are commonly seen in PTC.

**Methods:**

In total, 90 cases of PTC are randomly selected from archive paraffin blocks and 10μm sections were cut and processed for DNA extraction. *BRAF* ^*V600E*^ mutation and 8 types of *KRAS* mutations were investigated using Real Time PCR.

**Results:**

*BRAF* ^*V600E*^ mutation was identified in 46% of PTC while *KRAS* mutations were seen in 11% of PTC. There was significant correlation between *BRAF* ^*V600E*^ mutation and PTC larger than 5cm in diameter, positive surgical margin and lymph node metastasis. *BRAF* ^*V600E*^ mutation was significantly higher in patients with less than 55-year of age than those more than 55-year of age. *BRAF* ^*V600E*^ mutation was significantly higher in patients with family history of thyroid cancer than those without. There was no significant difference in *BRAF*^*V600E*^ mutation between males and females, PTC classic and follicular variants, unifocal and multifocal PTC. There was a significant higher percentage of *BRAF* ^*V600E*^ mutation in classic PTC than papillary microcarcinoma variant. There was no significant age, gender, histologic type, tumor size, lymph node metastasis, tumor focality, and surgical margin status differences between KRAS mutated and non-mutated PTC.

**Conclusion:**

*BRAF* ^*V600E*^ and *KRAS* mutation are seen in a significant number of PTC in the UAE. *BRAF* mutation is significantly correlated with large tumor size, positive surgical margins and lymph node metastasis suggesting an association between *BRAF* ^*V600E*^ mutation and tumor growth and spread.

## Introduction

Thyroid cancer is the most common endocrine malignancy and clinical thyroid cancer accounts for 1–2% of all cancers [1]. Papillary thyroid carcinoma (PTC) is the most common malignant thyroid neoplasm comprising 80–90% of all thyroid malignancies [2].

PTC has many histologic subtypes including, classical papillary, follicular, encapsulated, papillary microcarcinoma, columnar cell, diffuse sclerosing, tall cell, cribriform-morular, hobnail, PTC with fibromatosis, solid/trabecular, spindle cell, clear cell, warthin–like and oncocytic PTC [3].

The clinical behavior of PTC diverges widely, from non-aggressive microcarcinomas that grow very slowly and are usually associated with excellent prognosis to an aggressive widely invasive PTC with metastasis that can be fatal [4]. Molecular alterations in the sequence composition of cellular molecules such as DNA, RNA, and proteins usually precede the development of PTC. These alterations are frequently initiated by specific mutations in growth signal genes such as serine/threonine-protein kinase (BRAF) or Rat sarcoma viral oncogene homolog (KRAS) which will be translated into oncoproteins that lead to uncontrolled growth signals within affected follicular cells [5–7]. BRAF and KRAS mutations are commonly seen in PTC [5].

*BRAF*, which is one of the three *RAF* genes of serine/threonine kinases (*ARAF*, *BRAF*, and *CRAF*) is involved in growth signals transmission and it is the immediate downstream of *RAS* gene. *BRAF* is an important player of the mitogen-activated protein kinase (MAPK) pathway.

This pathway conveys the extracellular signals from various hormones, cytokines and growth factors to the nucleus through the activation of signal cascades. Normally, activation of receptor tyrosine kinase (RTK) leads to the dimerization of receptors and tyrosine residue phosphorylation, which activates RAS kinase. Then, RAS kinase activates the phosphorylation of RAF kinases, which in turn activate the dual-specificity protein kinases: MAP/extracellular signal-regulated kinase (MEK) 1 and 2. MEK1/2 phosphorylates and activates extracellular signal-regulated kinases (ERK) 1 and 2. ERK1/2 regulates various transcription factors involved in increased expression of genes involved in cell proliferation, differentiation and apoptosis [5].

Mutation in *BRAF* is seen in 29–69% of PTC, making *BRAF* mutations the most common demarcated molecular abnormality in PTC [8]. Carcinogenesis is a complex process involving complex interacting signaling pathways instead of a single linear stream of the MAPK pathway. The high frequency of *BRAF*^V600E^ mutation in tall-cell variant, an aggressive variant of PTC, suggests that *BRAF* ^V600E^ mutations might be associated with an aggressive phenotype [9]. Studies have shown that induction *of BRAF*^*V600E*^ expression in rat thyroid cells facilitated the acquisition of secondary genetic events through induction of genomic instability [10].

The RAS proteins are located on the cytoplasmic surface of the cell membrane. RAS proteins convey extracellular signals that promote the proliferation, differentiation, and survival of cells. [11]. The *RAS-RAF-MEK-ERK* pathway is activated in 30% of human cancer [12].

In PTC, functional mutation in RAS has been identified in 0–10% of Asian PTC [13]. RAS mutation can promote thyroid tumorigenesis through the *RAS-RAF-MEK-ERK* pathway or through its interaction with PI3K/AKT pathway [14].

Thyroid cancer is the 3^rd^ most common cancer among UAE citizens and the 2^nd^ most common cancer among females in the UAE [15]; hence identification of the molecular changes may have impact on the diagnosis and treatment of PTC. In this study, we evaluate the frequency of *BRAF* ^*V600E*^ and *KRAS* mutations in PTC and their correlation with clinical and pathological changes. This is the first study on *BRAF* ^V600E^ and *KRAS* mutations in PTC in the United Arab Emirates.

## Materials and methods

### Collection of specimens

In total, 90 formalin fixed paraffin-embedded (FFPE) tissue blocks from surgically removed thyroid specimens, during the period 2011–2016, were randomly collected from the Department of Pathology, Tawam Hospital, Al Ain, United Arab Emirates. Three-um sections were prepared from selected blocks and stained with hematoxylin and eosin (H&E) stain. All sections were examined microscopically by a pathologist who participates in this study to be sure that the sections contain significant area of PTC (>50%of neoplastic cells). One ten-um section containing tumor-rich areas was taken from each block and was put in a separate labeled Eppendorf tube. New blade was used in cutting each block to prevent tissue contamination from case to case.

The protocol of the present study conformed to the ethical guidelines of the World Medical Association, Declaration of Helsinki, and was approved by Al Ain Medical District Human Research Ethics Committee (THREC-438). Patients or their caregivers signed a written consent allowing using their anonymous material for research purposes.

### Histopathological classification of selected cases

H&E stained sections of selected cases were reviewed and classified according to the 4^th^ edition of WHO classification of tumors of thyroid gland [3] by a pathologist participated in this project. Tumors were called classic if they show predominant papillary growth with classic papillary nuclear features. Tumors were called papillary microcarcinoma if they show predominant papillary or follicular growth with classic papillary nuclear features and have a size of ≤1cm in greatest dimension. Tumors were called follicular variant if they show predominant follicular growth with classic papillary nuclear features. Follicular variant has two subtypes; the encapsulated with invasion, when the tumor is encapsulated and there is invasion of the capsule, while the infiltrative subtype when the tumor lack the capsule and shows infiltration of the stroma.

## Collection of demographic data of selected cases

The demographic data and the clinical information were extracted from the electronic medical files of the subjects with the identified papillary thyroid carcinoma. The collected data include age at diagnosis, gender, body mass index (BMI), tumour size, thyroiditis, focality, family history of thyroid cancer, exposure to external radiation, smoking and post surgical TNM staging.

### DNA isolation

Genomic DNA was isolated from each sampled tissue sections with the REPLI-g FFPE Kit (Qiagen, Hilden, Germany) for direct whole genome amplification of DNA from FFPE tissue according to the manufacturer’s instructions. Briefly, 1x FFPE lysis solution was prepared and 100 μl was added to the tissue section and mixed and centrifuge briefly. The samples were incubated at 95°C for 10 min to melt the paraffin followed by cooling down the sample to room temperature. Then 2μl of Proteinase K was added to each sample and mixed and centrifuge briefly. Each sample was then incubated for 60 min at 60°C and then for a further 10 min at 95°C. Then, each 10μl of the lysed tissue section was transferred into a new micro centrifuge tube. The FFPE master mix was prepared as per manufacturer instructions on ice and vortex and centrifuge briefly. Then, 10μl FFPE master mix was added to 10 μl DNA from the lysed tissue then mixed and centrifuged briefly. Then the samples were incubated at 24°C for 30 min. Then the reaction was stopped by incubation at 95°C for 5 min followed by cooling down to 4°C using a thermal cycler. Finally, the samples were incubated at 30°C for 8 h (high-yield reaction) then stopping the reaction by incubation at 95°C for 10 min. The amplified DNA was stored at -20°C until required for downstream applications.

### Quantification of DNA

Quantifiler^™^ Trio DNA Quantification, Kit Catalog number: 4482910 was used to quantify the total amount of amplifiable human DNA in the sample. For the Quantifiler^™^ Trio DNA Quantification Kit: the Quantifiler^™^ Trio Primer Mix and Quantifiler^™^ THP PCR Reaction Mix was mixed as per manufacturer’s instructions. The PCR mix was vortexed and centrifuged briefly. The 2 μL of gDNA was added to the applicable wells. The reaction plate was sealed with the Optical Adhesive Cover and care was taken to remove bubbles. The plate was centrifuged at 3,000 rpm for about 20 seconds in a tabletop centrifuge with plate holders to remove any bubbles. A total of 90 samples were processed for DNA isolation. The concentration of gDNA was determined using Nanodrop instrument using nuclease free water as blank solvent.

### Determination of *KRAS*/ BRAF mutation

GenoScreen *KRAS/BRAF* Real Time PCR Kit (DiagCor Bioscience Inc. Ltd, Hong Kong) was used for a qualitative assessment intended for the detection of eight *KRAS* mutations in codon 12 and 13, and one *BRAF* mutation in codon 600 using real-time PCR assay. The GenoScreen *KRAS/BRAF* Real Time PCR Kit is developed based on PCR amplification of mutant DNA with specific primers, detected by real time polymerase chain reaction (PCR) technology. Detection of target amplified product (amplicon) is achieved by the cleavage of dual fluorescent dye labeled oligonucleotide probes during the quantitation of mutant DNA.

### Procedure

All the PCR reagents were pipetted mix and spin down before use. The final PCR reaction (volume 10 μL) was prepared according to the manual. The recommended reaction component volumes to amplify DNA for *KRAS/BRAF* PCR Master Mix: 5μL, Primer Mix: 3μL, Template (DNA/ H2O): 2μL. From the 8 μL of PCR mixture (containing *KRAS/BRAF* PCR Master Mix and Primer Mix) aliquoted into each PCR reaction and added the appropriate amount of DNA template suggested and finally the reaction volume was top up to 10 μL with DNase Free Water if necessary. The mixture was then spin down and placed in real time PCR thermal cycler, QuantStudio3 (QS3). The reporter was select “FAM”, “NFQ-MGB” for Quencher and “ROX” for passive reference. Amplification Profile was set at 95°C, 10 min/1 cycle and amplification at 95°C, 15 sec/ 50cycle finally 60°C, 1 min FAM channel.

## Data analysis and interpretation

The data was analyzed after setting the threshold for FAM signals. The cycle threshold (Ct) value was set at 1/20 of each individual marker’s highest fluorescence point for the run (i.e. FAM). The FAM signal from the positive and negative assay was used to determine the validity of the real time run. The positive assay gave the Ct values between 24–38, and Negative Control assay was Ct value greater than 45. The results were determined using QS3 Real-Time PCR System. For each mutation assay the Ct value was used per the manufacturer’s instructions. For *KRAS* G12R< 35, *KRAS* G12S< 33, *KRAS* G12C < 34, *KRAS* S G12D < 35, *KRAS* G12A< 34, *KRAS* G12V < 32, *KRAS* G13C< 36, *KRAS* G13D< 35, *BRAF* ^V600E^ < 35, Positive Control 24–38, Negative Control> 45.

### Statistical analysis

The statistical analysis was computer assisted using SPSS for windows version 20 (SPSS Inc, Chicago, USA). Student’s t-test was used to compare continuous variables. Quantitative variables were analyzed with the chi-squared test and correlations of ordinal variables using the Spearman rank correlation coefficient and Chi-square (Fisher’s exact) test. P value <0.05 were considered significant. Where appropriate numerical data were presented as the mean ±SD.

## Results

### Demographic data

In total, 90 cases of PTC were analyzed in this study. The mean age was 41.21 ± 13.94, the mean BMI was 29.18 ± 6.01, and the female to male ratio was 2.33. Family history of thyroid cancer was seen in 13% of cases, while family history for other neuroendocrine tumors was seen in 1% of cases. History of exposure to external radiation was seen in 3% of cases. Only 8% of cases were smokers (Table 1).

**Table 1:** Demographic data of 90 cases of PTC.

Parameter	Mean ± SD (%)
Age	41.21 ± 13.94
BMI	29.18 ± 6.01
Male	27 (30.0%)
Female	63 (70.0%)
Family History of thyroid cancer	12(13%)
Family History of other neuro-endocrine tumors	1(1%)
History of Exposure to external radiation	3(3%)
Smoker	7(8%)

### Histologic types of PTC

Classic (conventional) PTC, which exhibits a predominant papillary pattern of growth with characteristic nuclear features of PTC, was the most common type comprising 46.6% (42) of the cases followed by microcarcinoma 30% (27) and follicular variant PTC comprising 23.4% (21).

Most of the papillary microcarcinomas 25 (93%) exhibit papillary pattern of growth similar to the classic PTC, and only 2 cases (7%) exhibit predominate follicular pattern of growth similar to follicular variant PTC. The follicular variant of PTC, which exhibit a predominant follicular pattern of growth with the characteristic nuclear features of PTC, has two subtypes. The infiltrative which comprises 45% (10) of the cases, and the encapsulated invasive subtype which comprises 55% (11) of the cases (Table 5).

## Histopathological features

### Site of involvement

Right lobe was the most common site of PTC comprising 48% (43) of cases, while left lobe and both lobes were involved in 28% (24) and 21.2% (22) of cases respectively (Table 2).

**Table 2:** Showing histopathologic features of 90 cases with PTC.

Features		Frequency
**Lobes**	Right	43 (48%)
	Left	25 (28%)
	Both	22 (24%)
**Focality**	Unifocal	58 (64%)
	Multifocal	32 (36%)
**Capsule**	No capsule	72 (80.0%)
	Encapsulated	18 (20%)
**Positive lymphovascular invasion**		13 (14%)
**Positive surgical margins**		22 (24%)
**Thyroiditis**		37 (41%)

### Focality

Unifocal PTCs were seen in 64% (58) of the cases while multifocal PTC was seen in 36% (32) of the cases (Table 2).

### Presence of capsule

**Fig 1:**
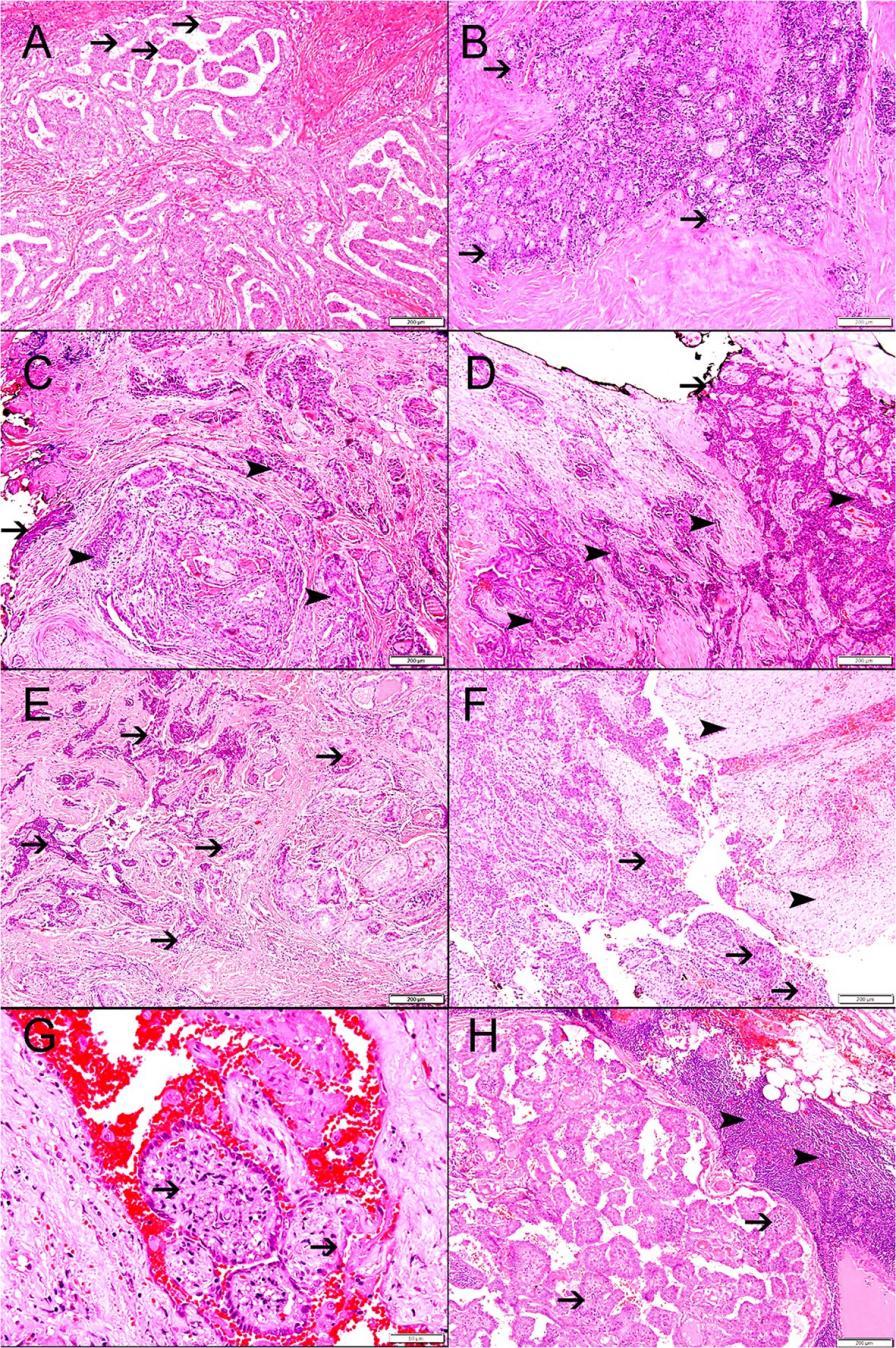


Most of PTC were non-encapsulated and comprising 80% (72) of cases while encapsulated PTCs were seen in 20% (18) of the cases (Table 2) (Fig 1).

### Lymphovascular Invasion (LVI)

Positive LVI was seen in 14% (13) of PTC, while unidentified LVI was seen in 86% (76) of the cases (Table 2) (Fig 1).

### Surgical margin involvement

Positive surgical margin was seen in 24% (22) of PTC, while free surgical margin was seen in 76% (68) of the cases (Table 2), (Fig 1).

### Lymphocytic thyroiditis

Lymphocytic thyroiditis was seen in 41% (37) of PTC (Table 2).

## TNM staging

### Tumor size

In total, 49 (54%) cases of PTC were diagnosed at T1, while 22 (24%), 17 (19%) and 2 (2%), were diagnosed at T2, T3, and T4 respectively (Table 3).

**Table 3:** Showing TNM staging system characteristics of 90 cases of PTC.

Parameter		Frequency	Percent
**T1**	Tumor ≤2 in greatest dimension, limited to the thyroid	49	54.4
**T2**	Tumor >2 cm, but ≤4 cm in greatest dimension, limited to thyroid	22	24.5
**T3**	Tumor >4 cm limited to the thyroid, or gross extrathyroidal extension invading only strap muscles	17	18.9
**T4**	Gross extrathyroidal extension beyond the strap muscles	2	2.2
**N1**	Metastasis to regional nodes	23	25.5
**M1**	Distant metastasis	1	1.1

### Lymph nodes involvements

In total 22 (24%) cases show N1, while 1 (1%), and 1 (1%) were N2 and N3 respectively. 66 (74%) of PTC have no lymph node metastasis (Table 3), (Fig 1).

### Distant metastasis

Only one cases of PTC shows distant metastasis (Table 3).

### Frequency of mutations

*BRAF* ^V600E^ mutation was seen in 46% (41) of PTC. *KRAS* mutations were seen in 12% (11) of PTC. In total, 8 different *KRAS* mutations at codon 12 and 13 0f *KRAS* were investigated. *KRAS*_G12V mutation was the most common *KRAS* mutation and was seen in 7% (6) of PTC, while *KRAS*_G12S, *KRAS*_G12D, *KRAS*_G13C and *KRAS*_G13D were seen in 2% (2), 1% (1), 1% (1), 1% (1) respectively. *KRAS*_G12A, *KRAS*_G12C and *KRAS*_G12R mutations were not identified in our samples (Table 4).

**Table 4:** Frequency of mutation.

Type of mutation	Papillary carcinoma (n = 90)
*BRAF_V600E*	41 (46%)
Any *KRAS* mutation	11 (12. %)
*KRAS*_G12V	6 (7%)
*KRAS*_G12S	2 (2%)
*KRAS*_G12D	1 (1%)
*KRAS*_G13C	1 (1%)
*KRAS*_G13D	1 (1%)
*KRAS*_G12A	Nil
*KRAS*_G12C	Nil
*KRAS*_G12R	Nil

## Correlation between *BRAF* ^V600E^ and *KRAS* mutations with demographic and histological parameters

### Age and *BRAF* ^*V600E*^ and *KRAS* mutations

*BRAF* ^*V600E*^ mutation was significantly higher in patients with less than 55-year of age than those higher than 55-year of age (*P = 0*.*014*) (Table 5). There was no signification correlation between *KRAS* mutation and any age group (Table 5).

**Table 5:** Correlation between BRAF and KRAS mutations and demographic and histological parameters.

	*KRAS* MUTATION			*BRAF* ^V600E^ MUTATION		
	Negative	Positive	*P-value*	Negative	Positive	*P-value*
	N (%)	N (%)		N (%)	N (%)	
Age						
<55 (N = 74)	66 (89.2)	8 (10.8)	0.385	38 (51.4)	36 (48.6)	0.014
>55 (N = 16)	13 (81.3)	3(18.8)		11(68.8)	5 (31.3)	
Gender						
Male (N = 27)	24(88.9)	3(11.1)	0.835	13(48.1)	14(51.9)	0.493
Female (N = 63)	55(87.3)	8(12.7)		36 (57.1)	27 (42.9)	
Family history of thyroid cancer	12(100)	0 (0)	0.329	3 (25)	9 (75)	0.036
History of smoking	7 (100)	0 (0)	1.00	3 (42)	4 (58)	0.702
Exposure to radiation	3 (100)	0 (0)	1.00	1 (33)	2 (66)	0.598
Histologic type						
Classic (N = 42)	38(90)	4 (10)		19 (45)	23(55)	
Microcarcinoma (N = 29)	22(81)	5(19)	0.434	17 (63)	10(37)	0.01
Follicular(N = 19)	19 (90)	2 (10)	1.00	13 (58)	8 (42)	0.08
Encapsulated with invasion(11)	11 (100)	0(0)		9 (82)	2(18)	
Infiltrative (10)	9 (90)	1(10)	1.00	6 (60)	4(40)	0.001
Tumor size						
T1(N = 49)	43 (87.8)	6(12.2)	0.994	31(63.3)	18(36.7)	0.052
T2(N = 22)	20(90.9)	2(9.1)	0.611	12(54.5)	10(45.5)	0.594
T3+T4(N = 19)	16(84.2)	3(15.8)	0.598	6(31.6)	13(68.4)	0.023
LN Metastasis						
Yes (N = 23)	20(87.0)	3(13.0)	0.572	6 (26)	17(74)	0.002
Focality						
Unifocal (N = 60)	52(86.7)	8(13.3)	0.653	34(56.7	26(43.3)	0.354
Multifocal (N = 30)	27(90.0)	3(10.0)		15(50.0)	15(50.0)	
Surgical margin						
Positive (N = 22)	19 (86.4)	3(13.6)	0.818	8(36.4)	14(63.6)	0.043
Lymphovascular invasion						
Yes (N = 13)	11(84.6)	2(15.4)	0.710	5(38.5)	8(61.5)	0.171

### Gender and *BRAF* ^*V600E*^ and *KRAS* mutations

There was no signification correlation between *BRAF* ^*V600E*^ mutation and any gender group as well as there was no signification correlation between *KRAS* mutation and any gender group (Table 5).

### Family history of thyroid cancer

There was a significant correlation between family history of thyroid cancer and *BRAF* ^*V600E*^ mutation (*P = 0*.*036*). There was no significant correlation between family history of thyroid cancer and *KRAS* mutation (Table 5).

### History of smoking

There was no significant correlation between history of smoking and *BRAF* ^*V600E*^ and *KRAS* mutation (Table 5).

### Exposure to radiation

There was no significant correlation between history of exposure to radiation and *BRAF* ^*V600E*^ and *KRAS* mutation (Table 5).

### Histologic variant and *BRAF* ^*V600E*^ and *KRAS* mutations

The percentage of *BRAF* ^*V600E*^ mutation was higher in classic PTC than follicular variant PTC but it did not reach the statistical significant (*P = 0*.*08*). In addition, there was no significant difference in the percentage of *KRAS* mutation between the classic PTC and the follicular variant PTC. (Table 5). There was a significantly higher percentage of *BRAF* ^*V600E*^ mutation in classic PTC than papillary microcarcinoma (*P = 0*.*0157*), while there was no significant differences in the percentage of *KRAS* mutation between classic PTC and papillary microcarcinoma.

### Tumor size and *BRAF* ^V600E^ and *KRAS* mutations

Tumor sizes above 4cm are significantly correlated with *BRAF* ^*V600E*^ mutation (*P = 0*.*023)*. Any tumor size was not correlated with any *KRAS* mutation (Table 5).

### Lymph node metastasis and *BRAF* ^*V600E*^ and *KRAS* mutations

There was a significant correlation between lymph node metastasis and *BRAF* ^*V600E*^ mutation (*P = 0*.*002*). There was no significant correlation between lymph node metastasis and *KRAS* mutation (Table 5) (Fig 1).

### Tumor focality and *BRAF* ^*V600E*^ and *KRAS* mutations

There was no signification correlations between *BRAF* ^*V600E*^ mutation and focality as well as there was no signification correlation between *KRAS* mutation and focality (Table 5).

### Surgical margin and *BRAF* ^*V600E*^ and *KRAS* mutations

There was a significant correlation between positive surgical margin and *BRAF* ^*V600E*^ mutation (*P = 0*.*43)*. There was no signification correlation between *KRAS* mutation and positive surgical margin (Table 5) (Fig 1).

### Lymphovascular invasion and *BRAF* ^*V600E*^ and *KRAS* mutations

There was no correlation between lymphovascular invasion and *BRAF* ^*V600E*^ or *KRAS* mutations (Table 5) (Fig 1).

## Discussion

Papillary thyroid carcinoma is the most prevalent type of thyroid cancer worldwide [16]. A lot of works have been done to identify fundamental mechanisms involved in the development of PTC [13, 14, 16–21]. Many studies have shown a constant rise in the incidence of PTC in different countries all over the world over the last decades [17]. Jung et al. have shown the increase in thyroid cancer incidence during the last four decades is accompanied by a high frequency of *BRAF* mutations and a sharp Increase in *RAS* mutations [18]. Identifying molecular changes in PTC is an important step in understanding major mechanisms participate in its development as well as open new doors for early diagnosis and treatment.

The MAPK pathway is an important intracellular signal transduction pathway that is required for maintaining cell proliferation, differentiation, and programed cell death in response to tyrosine kinase receptor (RTK) stimulation [19]. Moreover, it is a crucial player in the pathogenesis of PTC, as somatic mutations in its various components constantly drive the oncogenic process [20].

In this study we have identified *KRAS* mutation in 12% of PTC. We have identified 5 out of 8 investigated mutations in *KRAS gene*. Those mutations were identified in codon 12 and 13, and include KRAS_12V, *KRAS*_G12S, *KRAS*_G12D, *KRAS*_G13C and *KRAS*_G13D were seen in 7%, 2% (2), 1% (1), 1% (1), 1% (1) of PTC, respectively. In fact, to the best of our knowledge this is the first report of these mutations in *KRAS* gene in PTC using Real Time PCR.

In addition, there was no significant difference in the percentage of KRAS mutation between the classic PTC and the follicular variant PTC. This finding might be related to the low number of selected cases in this study.

The increase of the RAS mutations is explainable with a decrease of classic PTC variant and a sharp increase of follicular PTC variant in the last decades [18]. In our study, one quarter of the cases were pure follicular variant of PTC. Besides, the other three quarters were classic PTC, and although predominantly show classic papillary pattern, there are foci of follicular pattern as well seen in many of these PTCs, as part of histologic spectrum of classic PTC. This observation may explain the high rate of KRAS mutations in our study.

There are variable frequencies of *RAS* mutations in PTC. Goutas et al. have identified *KRAS* mutation in 54.5% of PTC while, Di Cristofaro et al. [22], Siraj et al. [20], Jung et al. [18] and Naito et al. [23] have shown *RAS* mutation in 25%, 8%, 14%, and 50% respectively.

Variability in frequencies of *RAS* mutations reflects differences in samples, method of DNA extraction, detection method of mutations, and possible geographical difference in the pattern of *RAS* mutation in PTC.

*RAS* mutation is considered as an early molecular event in follicular cell oncogenesis that leads to a well-differentiated neoplasm and may progress to a de-differentiated tumor following the gaining of further mutations [23, 24]. Knauf et al. have shown active *RAS* mutation can accelerate progression of cell cycle and promotes DNA damage by interfering with different cell cycle check points [24].

We have also shown *BRAF*^V600E^ mutation in 46% of PTC. Our finding has an intermediate position between Mediterranean countries and North American and some Far East countries [25–40] (Table 6). Some studies have close results to ours [28, 29, 30, 35, 36, 38, 39].

**Table 6:** Frequency *BRAF*^*V600E*^ mutation in different studies worldwide.

Study	*BRAF* ^V600E^ mutation %	Country
Rosenbaum et al. [23]	65	USA
Guan et al. [24]	62	USA
Wang et al. [25]	50	USA
Kebebew et al. [26]	49	USA
Frasca et al. [27]	39	ITALY
Lupi et al. [28]	44	ITALY
Elisei et al. [29]	37	ITALY
Fugazzola et al. [30]	32	ITALY
Costa et al. [31]	55	PORTUGAL
Zoghlami et al. [32]	43	FRANCE
Goutas et al. [19]	27	GREECE
Kim et al. [33]	73	KOREA
Ito et al. [34]	38	JAPAN
Langping et al. [35]	63	CHINA
Nelson etal. [36]	51	INDIA
TANG et al. [37]	50	TAIWAN
MARWA et al. [38]	55	EGYPT
Siraj et al. [18]	59	KINGDOM OF SAUDI ARABIA
Our study	46	UAE

The percentage of BRAF mutation was higher in classic PTC than follicular variant PTC but it did not reach the statistical significant (*P = 0*.*08*). It is possible that a higher number of included cases will improve the results. We think that this is a limitation in our study. In addition, we have shown a significantly higher percentage of *BRAF*^*V600E*^ in classic PTC than papillary microcarcinoma.

Moreover, we have also show a significantly higher percentage of *BRAF*^*V600E*^ mutation in infiltrative follicular variant of PTC than the encapsulate with invasion subtype of follicular variant of PTC. These results suggest that *BRAF*^*V600E*^ mutation is associated with tumor growth and spread [22, 33, 39].

Ugolini et al. have identified frequent *BRAF* ^V600E^ mutation in papillary thyroid microcarcinomas (PTMC) [41].

Knauf et al. have shown that *BRAF*^*V600E*^ transgenic mice develops poorly differentiated PTC with aggressive behavior which confirms the oncogenic role of *BRAF*^*V600E*^ mutation [42].

**Fig 2:**
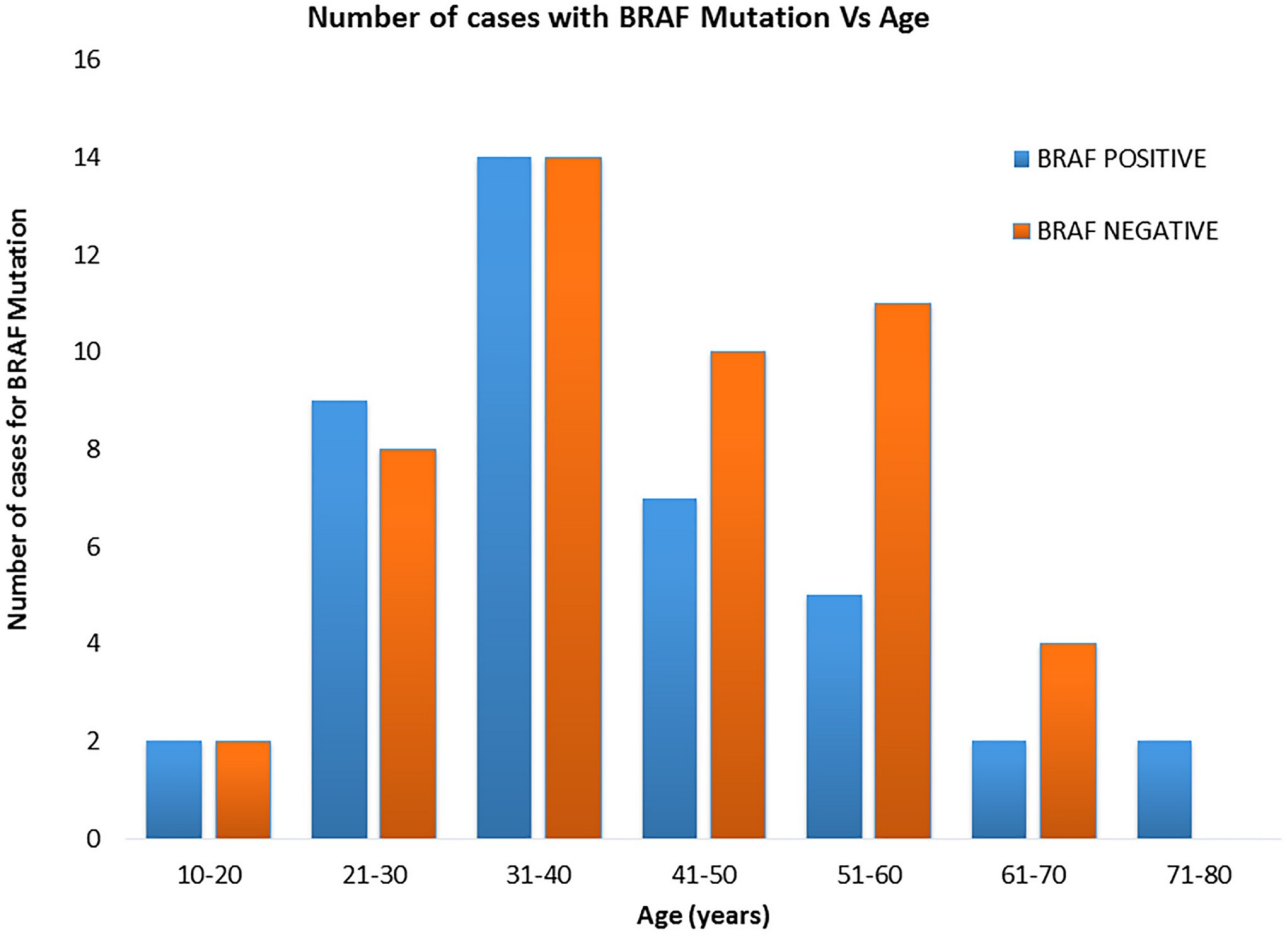


We also have shown a significantly higher frequency of *BRAF* ^*V600E*^ mutation in patients with ages younger than 55 years. Other studies [43–45] have shown a higher frequency *BRAF* ^*V600E*^ mutation among patients with ages higher than 55 years. We believe that this difference in the age pattern of *BRAF* ^*V600E*^ mutation is mainly due to the differences in patients samples between ours and these studies; as most of our PTC samples (70%) are from patients with ages younger than 55 years (Fig 2). In addition, a previous study in the UAE [16] have also shown more than three quarters of PTC cases were diagnosed below the age of 55 years. Geng et al. [46] have also shown a higher frequency of PTC with *BRAF* ^*V600E*^ mutation in pediatric age group of less than 10 years of age.

Moreover, we have shown a significant correlation between *BRAF* ^*V600E*^ mutation and PTC larger than 5cm in diameter and positive surgical margin suggesting an association between *BRAF* ^V600E^ mutation and an aggressive phenotype. This was also seen in other studies [47–50].

The association of a large size PTC with *BRAF* ^*V600E*^ mutation suggesting a critical rule of this mutation with cell proliferation. On the other hand, the clear associations of BRAF V600E mutation with positive surgical margin in this study points towards a crucial role of this mutation in increasing invasiveness of proliferating neoplastic cells. This was reported by Mesa et al., whom have shown conditional expression of *BRAF* ^V600E^ in thyroid cells markedly increased the Matrigel^™^ invasion of the transformed thyroid cells, which is more invasive than RET/PTC expressed cells [51].

We have shown a significant correlation between lymph node metastasis and *BRAF* ^V600E^ mutation, this finding supported by Carol et al. study [52] Lymphovascular invasion (LVI) is an important prognostic factor in in PTC and significantly associated with *BRAF* ^*V600E*^ mutation suggesting the presence of LVI should be considered as an indicator of aggressive clinicopathological features and patients with positive LVI should be followed up carefully for possible recurrence or metastasis [53]. In addition, *BRAF*^*V600E*^ mutation is an independent predictor of lymph node metastasis in PTC [54, 55].

## Conclusion

*BRAF* ^*V600E*^ and *KRAS* mutation are seen in a significant number of PTC in the UAE. *BRAF* mutation is significantly correlated with large tumor size, positive surgical margins and lymph node metastasis suggesting an association between *BRAF* ^*V600E*^ mutation and tumor growth and spread.
